# How do sleepwear and bedding fibre types affect sleep quality: A systematic review

**DOI:** 10.1111/jsr.14217

**Published:** 2024-04-16

**Authors:** Xinzhu Li, Mark Halaki, Chin Moi Chow

**Affiliations:** ^1^ Sydney School of Health Sciences, Faculty of Medicine and Health University of Sydney Camperdown New South Wales Australia; ^2^ Charles Perkins Centre University of Sydney Camperdown New South Wales Australia

**Keywords:** bedding, fibre, sleep, sleepwear

## Abstract

Sleepwear and bedding materials can affect sleep quality by influencing the skin and body temperature and thermal comfort. This review systematically evaluates the impact of sleepwear or bedding of different fibre types on sleep quality. A systematic search was conducted in six data bases plus Google Scholar and manual searches. Original articles that compared human sleep quality between at least two fibre types of bedding or sleepwear were included, resulting in nine eligible articles included in the review. The fibre types included cotton, polyester, wool, and blended materials for sleepwear; cotton, duck down, goose down, polyester and wool for duvet; and linen and a combination of cotton and polyester for bedding. The interplay between fibre materials and sleep quality is complex. Blended sleepwear demonstrated potential benefits for specific populations. Wool sleepwear showed benefits for sleep onset in adults (cool conditions) and in older adults (warm conditions). Linen bedsheets improved sleep quality under warm conditions in young adults. Goose down‐filled duvets increased slow‐wave sleep under cool conditions in young adults. However, a systematic comparison of fibre types is challenging due to the diverse nature of the studies evaluating sleep quality. Further research employing standardised methodologies with standard fibre samples in different populations and in different temperature conditions is imperative to elucidate comprehensively the effects of fibre choices on sleep quality. Despite the limitations and heterogeneity of the included studies, this analysis offers valuable insights for individuals seeking to optimise their sleep experiences and for manufacturers developing sleep‐related products.

## 1 INTRODUCTION

Sleep, accounting for approximately one‐third of an individual's lifespan, plays a fundamental role in maintaining human health and overall well‐being (Lee, 1997). Inadequate or poor sleep has been associated with detrimental effects on both cognitive and motor performance, mood regulation, as well as disruptions in metabolic, hormonal, and immunological systems (Ferrara & De Gennaro, 2001; Kecklund & Axelsson, 2016). The body temperature rhythms have a stable internal relationship with the sleep–wake cycle, as the timing of sleep is highly correlated with the phase of the body temperature rhythm (Monk, 1987). Sleep duration is extended when bedtime occurs on the falling limb of the core body temperature curve, whereas sleep duration is short when bedtime occurs on the rising limb of the curve. There is a strong tendency for sleep termination when sleep occurs at the temperature peak (acrophase) (Monk, 1987). Hence, sleep timing and duration are closely associated with changes in the circadian core body temperature.

Sleep quality can be affected by many factors such as health behaviours, physical health, psychological health (Xu et al., 2014), as well as environmental factors such as light, noise, temperature, air pollution, social neighbourhood safety, etc. (Johnson et al., 2018; Troynikov et al., 2018). Among the environmental factors, the thermal environment is very important for sleep maintenance (Lan et al., 2014; Lan et al., 2017; Troynikov et al., 2018). Previous studies have shown that an excessively high, low, or fluctuating air temperature can compromise sleep quality (Fletcher et al., 1999; Lan et al., 2014; Yao et al., 2007; Yao et al., 2011).

Sleepwear and bedding types can have an impact on sleep quality by affecting thermal comfort. Skin temperature plays an essential role in thermoregulation, as receptors for warmth and cold detection found in the skin help adjust peripheral blood flow and control heat loss (Krauchi et al., 2000; Xu & Lian, 2023). The skin's sensitivity to temperature fluctuations and the pervasive effect of ambient temperature on skin temperature have been well documented (Lan et al., 2014).

Sleep onset coincides with decreases in core body temperature, which is supported by selective vasodilation of distal skin regions to contribute to the decrease of core body temperature and promote sleep onset (Krauchi et al., 2000). During the sleep period, the skin temperature remains relatively stable, with a slight increase during rapid eye movement (REM) sleep (Okamoto‐Mizuno & Mizuno, 2012). Clothing provides thermal resistance and insulation for the human body, which is important for maintaining the thermal balance of the body during sleep.

Achieving thermal comfort plays a vital role in maintaining good sleep quality (Lan et al., 2017; Macpherson, 1973; Xu & Lian, 2023). Thermal comfort is a condition of mind that expresses satisfaction with the thermal environment (Auliciems & Szokolay, 1997) with a need to maintain a stable core body temperature (Nicol et al., 2012), in this way, it varies from person to person (Djongyang et al., 2010). The thermal exchange between a human body and the environment includes sensible heat loss from the skin, evaporative heat loss from the skin, and respiratory losses (Djongyang et al., 2010). A recent review by Xu and Lian (2023) highlighted the importance of designing a bedroom environment that promotes thermal comfort to improve sleep quality (Xu & Lian, 2023). The paper suggested that bedding conditions, such as the type of mattress and clothing, can impact thermal comfort and sleep quality. Sleepwear and bedding insulate the body and influence the skin and body temperature, and therefore can significantly affect sleep. Notably, the thermal properties of fabric fibres, including insulation and water vapour resistance, influenced by factors such as fibre type, thickness, and yarn structure, play a pivotal role. Specifically, natural fibres such as cotton and wool exhibit lower thermal conductivity when dry, but this can increase significantly after moisture absorption (Kothari, 2006). To optimise thermal comfort under normal or warm conditions, a fibre's water vapour permeability should be high to allow sweat to evaporate from the skin and to keep the skin dry (Kothari, 2006), for example, wool has a higher water vapour permeability than cotton and polyester, which allows efficient sweat evaporation, keeping the skin dry and enhancing thermal comfort (Bhatia & Malhotra, 2016).

Although there have been numerous studies investigating the effects of various fibre types of sleepwear and bedding on sleep quality, to the best of our knowledge, no systematic reviews have been conducted on this topic. Therefore, this review aims to systematically evaluate the impact of sleepwear or bedding of different fibre types on sleep quality by summarising the existing evidence. By analysing the effects of fibre types and thermal properties of sleepwear and bedding on sleep outcomes, this review aims to provide insights into selecting appropriate materials for better sleep quality.

## 2 METHOD

This systematic review was conducted in accordance with the recommendations outlined in the Preferred Reporting Items for Systematic Reviews and Meta‐analyses (PRISMA) statement (Page et al., 2021). The study protocol was registered with PROSPERO under the registration number CRD42021204652.

### 2.1 Study search

Systematic literature searches were conducted using six electronic databases (MEDLINE, Embase, CINAHL, Web of Science, Scopus, Proquest) and additional searches were conducted in Google Scholar with the first 17 pages and via citation searches. These data were added to the search results. An initial search was conducted on 18 August 2021 and updated every month by search alerts until November 2023. The search strategy contained four concepts with their combinations (bedding OR sleepwear) AND fibre AND sleep quality. For each concept, both text words and MeSH terms (controlled vocabulary) were searched where applicable.

The detailed keywords for each concept were as follows:

- bedding – “bedding and linen*” OR bed?sheet* OR “bed linen*” OR bedding* OR “quilt cover*” OR bedspread* OR “fitted sheet*” OR “top sheet*” OR “bed cover*” OR coverlet* OR comforter* OR quilt* OR duvet* OR blanket* OR underlay;
- sleepwear – pyjama* OR pyjama* OR cloth* OR night?dress* OR nighti* OR nightshirt* OR nightcloth* OR night?gown* OR Sleep?wear* OR “night garment*” OR lounge?wear;
- fibre – cotton* OR wool* OR “Merino wool*” OR flannelette* OR hemp OR viscose* OR modal* OR lycra* OR lyocell* OR polyester* OR polycotton* OR rayon* OR synthetic* OR silk* OR linen* OR bamboo OR textile* OR fibre* OR fibre* OR fabric* OR flax*;
- sleep quality – “sleep polysom*” OR “quality of sleep*” OR “sleep efficienc*” OR sleep* OR polysomnography* OR “Pittsburgh Sleep Quality Index” OR “PSQI” OR “Insomnia severity index” OR “ISI” OR “Insomnia index” OR Actigraph* OR “sleep assessment*” OR Polygraph* OR “Sleep Stage*” OR “sleep diar*” OR “sleep quality scale*” OR “sleep monitoring”.

The search included all studies that had an English title and abstract without other limitation. All results were exported into EndNote X9 for selection.

### 2.2 Study selection

After removing duplicates, all titles and abstracts were screened independently by two reviewers (XL and CMC or MH) for inclusion. Disagreements between individual judgements were resolved via discussion within the team. Studies were included in the review if they satisfied the following criteria: the study was conducted on humans of any age, reported sleep quality outcome measures (objective or subjective), investigated fibre type of the bedding or sleepwear, compared at least two fibre types (e.g., cotton vs wool). Studies were excluded if they only investigated one fibre type (e.g. cotton only), investigated the effect of chemical or medicinal fibre coatings, pillows, mattresses, or other supporting systems such as weighted blankets or clothes with different tightness. If an article potentially matched the inclusion criteria, the full text was reviewed using the same process and criteria. References where a full text could not be accessed were excluded (Lu et al., 2010). If separate references were examining the same study, the most updated data were included (He et al., 2019; Utkun, 2013). Articles written in languages other than English were translated via Google translate, and the accuracy of the translated information was corroborated by individuals proficient in the respective language.

### 2.3 Quality assessment

The methodological quality of each study was assessed using Joanna Briggs Institute (JBI) Critical Appraisal Tools (quasi experiments and randomised controlled trials). Two reviewers (CMC and XL) independently evaluated the quality of included articles. Disagreements in scoring were discussed until consensus was reached.

### 2.4 Data extraction and analysis

The following information was extracted from each study: (1) publication information: title, author(s), publish year, doi or url; (2) participants: number, age, sex, health conditions; (3) study design; (4) sleepwear, bedding, and fibre types; (5) sleep outcomes: measurement method (subjective or objective), parameters and data. Data presented in figures were extracted using GetData Graph Digitizer 2.26 software. XL extracted data and CMC or MH checked all extracted data. Disagreements between individual judgements were resolved via discussion within the team. For missing data, the corresponding author was contacted for unreported data. If unreachable, the data was recorded as missing (not reported, NR).

The data were analysed in Microsoft Excel. Computation of Hedges’ g effect sizes of differences in sleep parameters between fibre types was made to facilitate data interpretation, where Hedges’ g values of 0.20, 0.50, and 0.80 are considered to be indicative of small, medium, and large effect sizes (Cohen, 1992). A *p*‐value less than 0.05 is considered to indicate a statistically significant difference.

$${\text{Hedges}}^{\prime }g=\frac{M1-M2}{\sqrt{\frac{\left({{\mathrm{SD}}_{1}^{2}+\mathrm{SD}}_{2}^{2}\right)}{2}}}$$

M1 and M2 refer to the mean value of fibre 1 and fibre 2 respectively; SD1 and SD2 refer to the standard deviation of fibre 1 and fibre 2 respectively.

## 3 RESULTS

### 3.1 Study selection

**FIGURE 1:**
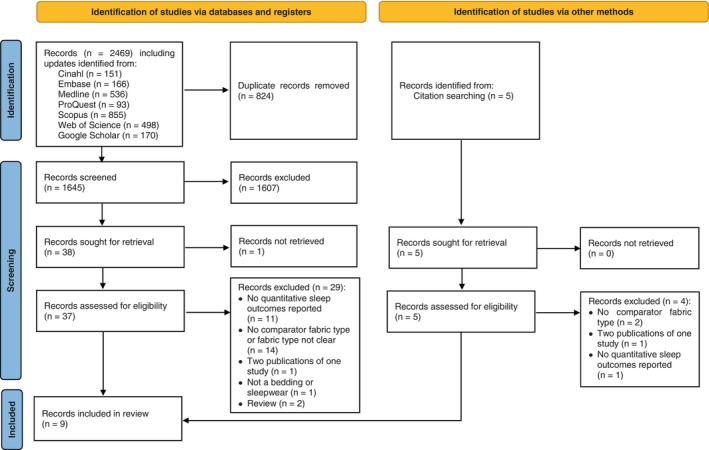
PRISMA flow chart of study selection in the systematic review.

The literature search across databases yielded 2362 references. After duplicates were removed, abstract and full text screening, nine studies (Araujo et al., 2013; Chow et al., 2019; He et al., 2019; Lee et al., 2004; Nejedlá & Minařík, 2016; Okamoto‐Mizuno et al., 2013; Okamoto‐Mizuno et al., 2015; Shin et al., 2016; Utkun et al., 2015) were included for the systematic review. A total of 25 studies were excluded by full text for: no quantitative sleep outcomes reported for nine records, no control fibre for nine records, no clear fibre details for three records, with more updated results in another publication for one record, no bedding or sleepwear for one record, or review articles for two records. Figure 1 represents the detailed PRISMA flow chart of the study screening and selection process. All the nine included articles were experimental studies. A detailed description of the included studies is given in Table 1.

**TABLE 1:** Summary of included studies.

Authors year country	Participants	Method				Outcomes (only statistically significant data are reported)
		Design	Ambient condition	Study procedures	Comparators	
(Lee et al., 2004) Republic of Korea	*N* = 9 0 male Age: 12 ± 2 years BMI: 24.3 ± 0.8 Healthy	Cross‐over, counter‐balanced	NR	3 consecutive days in sleep laboratory, 2 consecutive nights in cotton and 2 consecutive nights with experimental wear. (5 control, 4 MFF) 2‐week wash out then switch	Sleepwear control fibre: 100% cotton; MFF: super‐absorptive and fast‐drying capacities, anti‐bacterial, sun‐blocking, and far‐infrared radiation	**S1 (%)** Blended: 9.4 ± 3.1, Cotton: 15.2 ± 2.5, *p* < 0.05, Hedges’ *g* = −1.96 **S2 (%)** Blended: 36.1 ± 4.5, Cotton: 44.1 ± 9.3, *p* < 0.01, Hedges’ g = −1.02 **SWS (%)** Blended: 35.0 ± 2.9, Cotton: 18.4 ± 6.5, *p* < 0.01, Hedges’ g = 3.14 **subjective sleep quality on a VAS** (with 0 representing “feel very bad after sleep” and 100 representing “feel very good after sleep”) Blended: 83.2 ± 7.3, Cotton: 73.2 ± 8.2, *p* < 0.01, Hedges’ g = 1.22
(Araujo et al., 2013) Portugal	*N* = 18 61.1% male Age: 7 years BMI: NR All with atopic dermatitis (AD), 33.3% with personal history of atopy (Allergic rhinoconjunctivitis & asthma)	RCT	NR	From D0 the clothes were used continuously (24 h/day) for 7 days. After D7, the clothes were only used overnight, until D90. Clinical assessments taken at D0, D7, and D90. Participants slept in their own home	Babygrows (long sleeve no pants) for babies around 1‐year‐old and pyjamas (short top long pants) and sockets for older patients Trial group: 70% cotton fibres, 20% cellulose fibres with algae extracts and 10% silver activated algal cellulose fibres; control group: 100% cotton	**VAS of sleep disturbances** from 0 (lowest) to 10 (highest) points no significant differences were recorded
(Okamoto‐Mizuno et al., 2013) Japan	*N* = 8 100% male Age: 22.5 ± 3.5 years BMI: 20.3 ± 1.28 Healthy	Cross‐over, counter‐balanced	29 ± 0.5°C 70 ± 3%RH	The interval between the two conditions was between 2 and 6 days. Nap sleep study between 13:00 and 15:00	Sheet, bed pad and pillowcase: Cotton: cotton sheets, polyester bed pads and cotton pillowcases; Linen: linen (hemp) sheets, bed pads and pillowcases	**Wakefulness** (*N*) Linen: 7.9 ± 1.7, Cotton: 11.1 ± 2.5, *p* < 0.05, Hedges’ g = −1.42 **S1** (min) Linen: 17.8 ± 3.1, Cotton: 23.7 ± 3.9, *p* < 0.05, Hedges’ g = −1.58
(Okamoto‐Mizuno et al., 2015) Japan	*N* = 10 100% male Age: 23 ± 4 years BMI: 21.5 ± 2.1 Healthy	Cross‐over, counter‐balanced	29 ± 0.5°C RH: 70 ± 3%	Study conducted during August and September, before wearing, the sleepwears were conditioned at 5°C for at least 24 h. Crossover design for the two sleepwear types. The interval between the two conditions was between 2 and 6 days. Nap sleep study between 13:00 and 15:00	Sleepwears: short sleeve and long pants type C: 100% cotton; type L: polyamide‐based elastomer fibre 45%, rayon 55%	**S3 (min)** Cotton: 16.5 ± 3.3, Blended: 9.1 ± 3.1, *p* < 0.05, Hedges’ g = −2.21
(Utkun et al., 2015) Turkey	*N* = 8 50% male Age: 6–12 months BMI: NR HS: NR	Cross‐over, counter‐balanced	22–24°C RH: 40%–65%	1st, 3rd, 5th, 7th night: own underwear; 2nd, 4th, 6th, 8th night: test two‐piece pyjama set over own underwear: 2nd night D6; 4th night D6‐6, 6th night D2‐3; 8th night Ö4. Infant's sleep time, wakeup times during the night, duration of being awake, and wakeup time were followed by the mother	Two‐piece pyjama set: top and bottom, long sleeve D6, D6‐6, Ö4: 100% cotton with different weaves D2‐3: Blended (50% cotton +25% Tencel LF® + 25% Bamboo)	**Average sleep duration (min):** Cotton (D6): 659 ± 60.822, Cotton (D6‐6): 715 ± 46.664, Cotton (Ö4): 665 ± 82.412 Blended (D2‐3): 700 ± 77.068, ANOVA main effect *p* = 0.037 Hedges’ g: Blended (D2‐3) vs Cotton (D6): 0.56 Blended (D2‐3) vs Cotton (D6‐6): −0.22 Blended (D2‐3) vs Cotton (Ö4): 0.41
(Nejedlá & Minařík, 2016) Czech Republic	*N* = 1 male Age: young BMI: NR Healthy	single case	NR	The test was carried out on two defined days during 3 weeks, in total six measurements. Time in bed: 10 pm ‐ 6 am	Sleepwear: 100% cotton vs 60% TencelC +40% Tencel+PADh	**PSG** no significant differences were recorded
(Shin et al., 2016) Australia	*N* = 17 58.8% male Age: 24.6 ± 6.9 years BMI: 23.7 ± 2.2 Healthy (all females were on contraceptives)	Randomised, cross‐over, counter‐balanced	17.4°C ± 0.3°C and 22.4°C ± 0.5°C RH: 60.3% ± 2.0%	1 familiarisation night +8 nonconsecutive test nights no more than a week apart	Sleepwear (long sleeve and long pants): 100% cotton vs 100% Merino wool Duvet: wool vs polyester	**SOL (min)** wool sleepwear: 11.0 ± 8.2, cotton sleepwear: 15.0 ± 18.0, *p* = 0.043, Hedges’ g = −0.28 **SOL (at the ambient condition of 17°C)** wool sleepwear: 9.9 ± 6.6 min, cotton sleepwear: 18.1 ± 0.9 minutes, *p* = 0.006, Hedges’ g = −1.70 **%N3 at 22°C** wool sleepwear: 18.0 ± 1.2, cotton sleepwear: 19.6 ± 1.2, *p* < 0.05, Hedges’ g = −1.30
(Chow et al., 2019) Australia	*N* = 36 50% male Age: 60.0 ± 6.2 years BMI: 25.6 ± 4.1 Healthy (female participants were tested on the follicular phase)	Randomised, cross‐over, counter‐balanced	30.1 ± 0.5°C RH: 50.2 ± 2.9%	Study process: 1 adaptation night +3 testing nights randomly slept in cotton, polyester or wool sleepwear	Sleepwear: long sleeve and pants, loose‐fitting: cotton vs wool vs polyester	**SOL (min)** Cotton: 18.5 ± 23.5; Polyester: 18.2 ± 15.5; Wool: 16.0 ± 15.5, ANOVA main effect *p* = 0.04 Hedges’ g: Polyester vs Cotton: −0.01 Wool vs Cotton: −0.12 Wool vs Polyester: −0.14 SOL (min) in Old age subgroup (≥65 years, *n* = 13) Cotton: 26.7 ± 36.1 Polyester: 21.6 ± 21.0 Wool: 12.4 ± 13.4, ANOVA main effect *p* = 0.001 (*p* = 0.011 for difference between wool and cotton, *p* = 0.011 for difference between wool and polyester) Hedges’ g: Polyester vs Cotton: −0.17 Wool vs Cotton: −0.51 Wool vs Polyester: −0.51 **SFI (number. hour** ^**−1**^ **)**, sleep fragmentation index Cotton: 13.3 ± 5.8; Polyester: 13.7 ± 4.4*; Wool: 12.1 ± 4.2*, ANOVA main effect *p* = 0.01, (*p < 0.05 for difference between polyester and wool) Hedges’ g: Polyester vs Cotton: 0.08 Wool vs Cotton: −0.23 Wool vs Polyester: −0.37
(He et al., 2019) China	*N* = 8 50% male Age: 23 ± 3 years BMI: 21.8 ± 2.1 Healthy	Cross‐over, counter‐balanced	11.52 ± 0.85°C RH: 58.91 ± 7.52%	Every day, 2 subjects (1 male and 1 female) participated in the experiment. For each subject, three kinds of quilts were applied respectively to three‐night sleeping test in a fixed sleep chamber. The experimental conditions were presented in Latin‐square design. Time in bed: 23:30 pm – 7:30 am	Quilts (150 *210 cm^2^) with cotton cover: 90% white duck down, 90% white goose down or 100% cotton	**Subjective sleep quality** (5‐point scale, −2 very uncomfortable to +2 very comfortable) duck down: −0.03 ± 0.35, goose down: 0.48 ± 0.25, cotton: −0.25 ± 0.44, ANOVA main effect *p* < 0.05 (*p* = 0.077 for difference between duck down and goose down, *p* < 0.05 for difference between cotton and goose down) Hedges’ g: Duck down vs Cotton: 0.52 Goose down vs Cotton: 1.93 Goose down vs Duck down: 1.59 **PSG: N3%** duck down: 28.35 ± 1.07, goose down: 29.58 ± 0.91, cotton: 26.05 ± 1.23 (*p* = 0.083 for difference between duck down and cotton, *p* < 0.01 for difference between cotton and goose down) Hedges’ g: Duck down vs Cotton: 1.89 Goose down vs Cotton: 3.08 Goose down vs Duck down: 1.17

Abbreviations: HS, health status; NR, not reported; RH, relative humidity; SE, sleep efficiency; SFI, sleep fragmentation index; SOL, sleep onset latency; SWS, slow wave sleep; TST, total sleep time; VAS, Visual Analogue Scale; W, awake.

*Note*: N1/S1, sleep stage NREM stage 1; N2/S2, sleep stage NREM stage 2; N3, sleep stage NREM Stage 3; the merging of stage S3 and S4. N1/N2/N3 are standard from AASM from 2005 (AASM, 2005), S1/S2/S3/S4 are standard from Rechtschaffen and Kales (R&K) from 1968 (Rechtschaffen & Kales, 1968).

### 3.2 Risk of bias of included studies

In adherence to the CONSORT statement (Dwan et al., 2019), crossover studies of randomised design are an extension of RCT, therefore the risk‐of‐bias of three studies (Araujo et al., 2013; Chow et al., 2019; Shin et al., 2016) was evaluated using the JBI‐RCT tool. The rest of the studies (He et al., 2019; Lee et al., 2004; Nejedlá & Minařík, 2016; Okamoto‐Mizuno et al., 2013; Okamoto‐Mizuno et al., 2015; Utkun et al., 2015) were assessed using the JBI quasi‐experimental tool. The ranking results are presented in Tables 2 and 3.

**TABLE 2:** Joanna Briggs Institute (JBI) Critical Appraisal Tools for RCT studies.

JBI‐RCT	(Araujo et al., 2013)	(Shin et al., 2016)	(Chow et al., 2019)
1. Was true randomisation used for assignment of participants to treatment groups?	Yes	Yes	Yes
2. Was allocation to groups concealed?	Yes	Yes	Yes
3. Were treatment groups similar at the baseline?	Yes	Yes	Yes
4. Were participants blind to treatment assignment?	Yes	Not clear	Not clear
5. Were those delivering treatment blind to treatment assignment?	Yes	Not clear	Not clear
6. Were outcomes assessors blind to treatment assignment?	Not clear	Yes	Yes
7. Were treatment groups treated identically other than the intervention of interest?	Yes	Yes	Yes
8. Was follow up complete and if not, were differences between groups in terms of their follow up adequately described and analysed?	Not clear	Not applicable	Not applicable
9. Were participants analysed in the groups to which they were randomised?	Yes	Yes	Yes
10. Were outcomes measured in the same way for treatment groups?	Yes	Yes	Yes
11. Were outcomes measured in a reliable way?	Yes	Yes	Yes
12. Was appropriate statistical analysis used?	yes	Yes	Yes
13. Was the trial design appropriate for the topic, and any deviations from the standard RCT design accounted for in the conduct and analysis?	Yes	Yes	Yes

**TABLE 3:** Joanna Briggs Institute (JBI) Critical Appraisal Tools for quasi experimental studies.

JBI‐quasi‐experiment	(Lee et al., 2004)	(Okamoto‐Mizuno et al., 2013)	(Okamoto‐Mizuno et al., 2015)	(Utkun et al., 2015)	(Nejedlá & Minařík, 2016)	(He et al., 2019)
1. Is it clear in the study what is the “cause” and what is the “effect” (i.e. there is no confusion about which variable comes first)?	Yes	Yes	Yes	Yes	Yes	Yes
2. Were the participants included in any comparisons similar?	Yes	Yes	Yes	Yes	Yes	Yes
3. Were the participants included in any comparisons receiving similar treatment/care, other than the exposure or intervention of interest?	Yes	Yes	Yes	Yes	Yes	Yes
4. Was there a control group (control treatment)?	Yes	Yes	Yes	Yes	Yes	Yes
5. Were there multiple measurements of the outcome both pre and post the intervention/exposure?	Yes	No	No	No	Yes	No
6. Was follow up complete and if not, were differences between groups in terms of their follow up adequately described and analysed?	Not applicable	Not applicable	Not applicable	Not applicable	Not applicable	Not applicable
7. Were the outcomes of participants included in any comparisons measured in the same way?	Yes	Yes	Yes	Yes	Yes	Yes
8. Were outcomes measured in a reliable way?	Yes	Yes	Yes	Yes	Yes	Yes
9. Was appropriate statistical analysis used?	Yes	Yes	Yes	Yes	No	Yes

Nejedlá's (Nejedlá & Minařík, 2016) study only included one participant and statistical analysis could not be performed. Four studies (He et al., 2019; Okamoto‐Mizuno et al., 2013; Okamoto‐Mizuno et al., 2015; Utkun et al., 2015) did not apply multiple measurements on the outcomes. The rest of the studies (Araujo et al., 2013; Chow et al., 2019; Lee et al., 2004; Shin et al., 2016) were considered to have “good” methodological quality. These studies were characterised by robust controls, meticulous experimental design, and comprehensive statistical analyses, collectively underscoring their methodological rigour and capacity to yield reliable insights.

### 3.3 Study characteristics

The geographical distribution of the included studies reflects a diverse global perspective. Notably, two studies (Chow et al., 2019; Shin et al., 2016) were conducted in Australia, three studies (Araujo et al., 2013; Nejedlá & Minařík, 2016; Utkun et al., 2015) were conducted within the European region, while four studies (He et al., 2019; Lee et al., 2004; Okamoto‐Mizuno et al., 2013; Okamoto‐Mizuno et al., 2015) were conducted in East and Southeast Asia. As we included human studies covering the lifespan, the participants’ age ranged from 6 months to 66 years: three studies investigated infants (Utkun et al., 2015) and children (Araujo et al., 2013; Lee et al., 2004) (age < 18 years); five studies investigated adults (age: 18–50 years) (He et al., 2019; Nejedlá & Minařík, 2016; Okamoto‐Mizuno et al., 2013; Okamoto‐Mizuno et al., 2015; Shin et al., 2016); and one study investigated older adults (age: 50–70 years) (Chow et al., 2019). There were 115 participants (66 males and 49 females) included in total; one study included all female participants (Lee et al., 2004), three studies included all male participants (Nejedlá & Minařík, 2016; Okamoto‐Mizuno et al., 2013; Okamoto‐Mizuno et al., 2015) and five studies included both genders (Araujo et al., 2013; Chow et al., 2019; He et al., 2019; Shin et al., 2016; Utkun et al., 2015). In addition, one study investigated participants with a skin condition (atopic dermatitis) (Araujo et al., 2013), others were all healthy participants. Of all the product types, seven were sleepwear (Araujo et al., 2013; Chow et al., 2019; Lee et al., 2004; Nejedlá & Minařík, 2016; Okamoto‐Mizuno et al., 2015; Shin et al., 2016; Utkun et al., 2015), two included quilt filler (He et al., 2019; Shin et al., 2016), and one included bedsheets (Okamoto‐Mizuno et al., 2013).

Cotton emerged as the fibre type commonly used either as a control or an investigated fibre. The fibre types of sleepwear include cotton, wool, polyester, and five different types of blended materials (Araujo et al., 2013; Lee et al., 2004; Nejedlá & Minařík, 2016; Okamoto‐Mizuno et al., 2015; Utkun et al., 2015) (three studies with cellulose‐based fibres made from natural sources (Araujo et al., 2013; Nejedlá & Minařík, 2016; Utkun et al., 2015), one with synthetic materials of polyester Healtha and polyolefin (Lee et al., 2004), and one with natural and synthetic materials (Okamoto‐Mizuno et al., 2015)). Two studies (He et al., 2019; Shin et al., 2016) compared duvet fibre type between wool and polyester and between cotton and down fibres separately. One study compared bedsheets (Okamoto‐Mizuno et al., 2013) fibre type between linen and a combination of cotton bed sheet and polyester bed pad. Detailed material properties are displayed in Table 4.

**TABLE 4:** Summary of fibre material properties used in the included articles

Product	Author, year	Fibre component	Weight (g∙m^−2^)	Thickness (mm)	Water vapour permeability/moisture transmission (g∙m^−2^∙24 h^−1^)	Vapour resistance (m^2^∙Pa∙W^−1^)	Water spreading transport capacity	Air permeability/air transmission (l m^−2^∙s^−1^)
Sleepwear	(Lee et al., 2004)	Standard	NP	NP	‐	‐	NP	‐
		100% cotton	165	0.7	‐	‐	0.5	‐
		Polyester Healtha & Polyolefin	168	0.71	‐	‐	1.5	‐
	(Araujo et al., 2013)	‐	‐	‐	‐	‐	‐	‐
	(Okamoto‐Mizuno et al., 2015)	Standard	NP	NP	JIS L 1099 A	‐	‐	JIS L 1096 A
		100% cotton	100	0.42	10675.2^Δ^	‐	‐	1148^Δ^
		polyamide‐based elastomer fibre 45%, rayon 55%	160	0.18	10560^Δ^	‐	‐	104^Δ^
	(Utkun et al., 2015)	Standard	SFS 3192:1974 standard	SFS‐EN ISO 5084:1997 standard	Gore cup method	‐	‐	SFS‐EN ISO 9237:1996
		D2‐3: 50% cotton, 25% Tencel LF®,25% Bamboo	142	0.61	4975	‐	‐	1345
		D6: 100% cotton, structure: Plain weave	69.9	0.30	5643	‐	‐	1610
		D6‐6: 100% cotton, first‐type modified twill weave	79.4	0.49	4961	‐	‐	2780
		Ö4: 100% cotton, interlock knitted	216	0.80	4663	‐	‐	390
	(Nejedlá & Minařík, 2016)	‐	‐	‐	‐	‐	‐	‐
	(Shin et al., 2016)	Standard	NP	NP	‐	NP	‐	NP
		Cotton sleepwear: 100% cotton	153.80 ± 0.45	0.51 ± 0.00	‐	3.532 ± 0.07	‐	181.70 ± 7.32
		Wool sleepwear: 100% wool	161.40 ± 0.89	0.41 ± 0.00	‐	2.820 ± 0.06	‐	347.74 ± 7.32
	(Chow et al., 2019)	Standard	NP	NP	‐	‐	‐	‐
		100% Cotton	140.0 ± 0.0	0.57 ± 0.03	‐	‐	‐	‐
		100% Wool	143.5 ± 2.1	0.52 ± 0.01	‐	‐	‐	‐
		100% polyester	150.5 ± 0.7	0.49 ± 0.04	‐	‐	‐	‐
Bedsheets	(Okamoto‐Mizuno et al., 2013)	Standard	NP	NP	JIS L 1099	‐	‐	‐
		sheet linen 100%	234.6	0.44	480^Δ^	‐	‐	‐
		bed pad 100% linen	1345.1	14.8	302.4^Δ^	‐	‐	‐
		sheet 100% cotton	123.5	0.2	528^Δ^	‐	‐	‐
		bed pad 100% polyester	1245	24	307.2^Δ^	‐	‐	‐
Duvets and quilts	(Shin et al., 2016)	Standard	NP	NP	‐	NP	‐	‐
		Wool	694	15.7 ± 1.0	‐	60.09	‐	‐
		Polyester	933	17.3 ± 7.6	‐	20.51	‐	‐
	(He et al., 2019)	Standard	NP	NP	‐	‐	‐	‐
		100% cotton	133.2	4.18	‐	‐	‐	‐
		90% white duck down	200	8.7	‐	‐	‐	‐
		90% white goose down	Air density 200	8.7	‐	‐	‐	‐

Product	Author, year	Thermal resistance (m^2^·K∙W^−1^)	Thermal conductivity (W∙m^−1^·K^−1^)	Thermal insulation value (%)	Clo value cloth insulation (clo)	Thermal character q‐max (W∙cm^−2^)	Moisture regain (%)
Sleepwear	(Lee et al., 2004)	‐	NP	‐	‐	‐	‐
		‐	19.76^Δ^	‐	‐	‐	‐
		‐	6.97^Δ^	‐	‐	‐	‐
	(Araujo et al., 2013)	‐	‐	‐	‐	‐	‐
	(Okamoto‐Mizuno et al., 2015)	‐	‐	ThermoLaboII	‐	NP	gravimetric method under 27°C RH90%
		‐	‐	23.1	‐	0.15	12.5
		‐	‐	13.1	‐	0.27	15.8
	(Utkun et al., 2015)	Alambeta (manufactured by Czech SENSORA Company	Alambeta (manufactured by Czech SENSORA Company	‐	‐	‐	‐
		0.019	0.047	‐	‐	‐	‐
		0.01	0.04	‐	‐	‐	‐
		0.01	0.04	‐	‐	‐	‐
		0.02	0.07	‐	‐	‐	‐
	(Nejedlá & Minařík, 2016)	‐	‐	‐	‐	‐	‐
	(Shin et al., 2016)	NP	‐	‐	‐	‐	‐
		0.021 ± 0.0001	‐	‐	‐	‐	‐
		0.023 ± 0.0032	‐	‐	‐	‐	‐
	(Chow et al., 2019)	NP	‐	‐	‐	‐	‐
		0.030	‐	‐	‐	‐	‐
		0.025	‐	‐	‐	‐	‐
		0.030	‐	‐	‐	‐	‐
Bedsheets	(Okamoto‐Mizuno et al., 2013)	‐	‐	ThermoLaboII	‐	KES	‐
		‐	‐	36.4	‐	0.12	‐
		‐	‐	80.7	‐	0.09	‐
		‐	‐	29.0	‐	0.12	‐
		‐	‐	85.9	‐	0.12	‐
Duvets and quilts	(Shin et al., 2016)	NP	‐	‐	‐	‐	‐
		0.54	‐	‐	‐	‐	‐
		0.38	‐	‐	‐	‐	‐
	(He et al., 2019)	‐	NP	‐	NP	‐	‐
		‐	4.5	‐	1.433	‐	‐
		‐	1.102	‐	7.583	‐	‐
		‐	1.102	‐	7.583	‐	‐

*Note*: Entries marked with ^Δ^ have been converted from their original units. NP: standard used to obtain the measure was not provided.

Of the included studies, seven reported the bedroom ambient conditions. For the room temperature, two studies (He et al., 2019; Shin et al., 2016) were conducted below 20°C, two studies (Shin et al., 2016; Utkun et al., 2015) were between 21 and 25°C, and three studies (Chow et al., 2019; Okamoto‐Mizuno et al., 2013; Okamoto‐Mizuno et al., 2015) at 29–30°C. Most studies that reported relative humidity (RH) were conducted at a RH between 40% and 65%, except for two studies (Okamoto‐Mizuno et al., 2013; Okamoto‐Mizuno et al., 2015) which were conducted at a RH of 70%.

Two studies (Okamoto‐Mizuno et al., 2013; Okamoto‐Mizuno et al., 2015) were daytime nap sleep studies of a fixed time of 2 h while all others were overnight sleep studies, of which, three studies (He et al., 2019; Lee et al., 2004; Nejedlá & Minařík, 2016) had a fixed sleep time of 8 hours, with the others being sleep ad libitum. Of the nine studies, one study (Araujo et al., 2013) was conducted at the participants’ own home, whereas all other studies were conducted in a sleep laboratory.

Eight studies used objective measurements (actigraphy and/or polysomnography) while one study (Utkun et al., 2015) only used a sleep diary to assess sleep quality. The main sleep outcomes reported included sleep efficiency (SE) in six studies (Chow et al., 2019; Lee et al., 2004; Nejedlá & Minařík, 2016; Okamoto‐Mizuno et al., 2013; Okamoto‐Mizuno et al., 2015; Shin et al., 2016), total sleep time (TST) in five studies (Chow et al., 2019; Lee et al., 2004; Okamoto‐Mizuno et al., 2015; Shin et al., 2016; Utkun et al., 2015), proportion of sleep stages in six studies (Chow et al., 2019; He et al., 2019; Lee et al., 2004; Nejedlá & Minařík, 2016; Okamoto‐Mizuno et al., 2013; Okamoto‐Mizuno et al., 2015), sleep onset latency (SOL) and REM sleep and latency in five studies (Chow et al., 2019; Nejedlá & Minařík, 2016; Okamoto‐Mizuno et al., 2013; Okamoto‐Mizuno et al., 2015; Shin et al., 2016), wake after sleep onset (WASO) in four studies (Chow et al., 2019; Nejedlá & Minařík, 2016; Okamoto‐Mizuno et al., 2013; Shin et al., 2016) and Pittsburgh Sleep Quality Index (PSQI) score in two studies (He et al., 2019; Shin et al., 2016). Other sleep outcomes reported include the sleep fragmentation index (Chow et al., 2019), arousal index, subjective rated sleep quality (Lee et al., 2004), sleep disturbance (Araujo et al., 2013), and sleep comfort (He et al., 2019), each with one report.

### 3.4 Fibre properties

Seven articles (Chow et al., 2019; He et al., 2019; Lee et al., 2004; Okamoto‐Mizuno et al., 2013; Okamoto‐Mizuno et al., 2015; Shin et al., 2016; Utkun et al., 2015) provided the texture properties of the sleepwear, bedding, or duvet used in the study. A summary of the material properties is provided in Table 4.

### 3.5 Sleep outcomes using different fibres

#### 3.5.1 Sleepwear

##### Cotton vs blended materials

Five studies (Araujo et al., 2013; Lee et al., 2004; Nejedlá & Minařík, 2016; Okamoto‐Mizuno et al., 2015; Utkun et al., 2015) compared the sleep outcome between blended sleepwear and cotton sleepwear, and only Lee's study (Lee et al., 2004) reported that the blended sleepwear (Polyester Healtha & Polyolefin) promoted significantly shorter N1% and N2%, longer SWS% and a higher subjective sleep quality on VAS compared with cotton sleepwear. Although Okamoto‐Mizuno's study (Okamoto‐Mizuno et al., 2015) reported a significant difference in Stage 3, there were no significant differences in the combined stage 3 + stage 4, or other sleep variables. None of the other studies did reported any significant differences in sleep outcomes between cotton and blended sleepwear (Araujo et al., 2013; Nejedlá & Minařík, 2016; Utkun et al., 2015).

Utkun et al. (2015) compared infant sleep quality when wearing underwear made of cotton fibres with three different weave structures and those of blended materials (50% cotton +25% Tencel LF® + 25% Bamboo). No consistent finding was found for sleep outcome between cotton and blended sleepwear. It was found that the D2‐3 showed a medium positive effect on TST compared with D6 and a small positive effect compared with Ö4, which indicated that D2‐3 promoted a longer total sleep time than D6 and O4.

Nejedlá and Minařík (2016) reported non‐significant differences in sleep quality between 100% cotton sleepwear and blended sleepwear (60% TencelC /40% Tencel+PADh) across three nights each in one healthy young man, although participants reported the blended sleepwear was nicer and finer for sensation. Of the six sleep study nights, the participant's sleep was disrupted by a siren on one night. Statistical analysis was not conducted.

Araujo et al. (2013) reported non‐significant differences in sleep outcomes between biofunctional textile (consisting of 70% cotton fibres, 20% cellulose fibres with algae extracts, and 10% silver activated algal cellulose fibres) and all‐cotton sleepwear.

A nap study investigated the effects of pyjamas made of cotton and blended material (45% polyamide‐based elastomer fibre and 55% rayon) on sleep under mild humid heat exposure (Okamoto‐Mizuno et al., 2015) and reported a significant reduction with a large effect in Stage 3 when sleeping in the blended pyjama compared with sleeping in cotton (*p* < 0.05), while there were no significant differences in SWS (stage 3 + stage 4) and other sleep variables between the blended pyjamas and cotton pyjamas.

##### Cotton vs polyester

Chow et al. (2019) conducted a study to determine the influences of sleepwear made of cotton, wool and polyester on sleep quality for adults aged 50–70 years old, with different BMI (>25 vs. ≤25 kg·m^−2^), and PSQI (poor sleepers vs. good sleepers whose PSQI score ≤5). When comparing cotton and polyester, there were no significant differences reported for all the sleep parameters. However, with the interaction between sleepwear and PSQI group, poor sleepers had a significantly longer REM sleep latency when sleeping in polyester than in cotton (*p* = 0.037).

##### Cotton vs wool

Two studies compared Merino wool sleepwear (Chow et al., 2019; Shin et al., 2016) with cotton sleepwear with both showing that wool sleepwear promoted shorter SOL than cotton sleepwear.

Shin et al. (2016) conducted a study among young adults which evaluated sleepwear, bedding at two temperature conditions (Shin et al., 2016). The results showed that wool sleepwear produced a significantly shorter SOL than cotton sleepwear with a small effect overall, with a large effect at 17°C. However, a marginal significant interaction was observed under 22°C that sleeping in cotton produced more N3% than wool with a large effect. All the rest of the sleep parameters were not significantly different between cotton and wool sleepwear.

A study conducted among older adults (Chow et al., 2019) also showed a similar result that sleepwear significantly reduced the SOL compared with cotton (*p* = 0.044) with no effect. Subgroup analysis showed significant differences in the following parameters: for age > 65 years old, SOL was significantly reduced when sleeping in wool compared with sleeping in cotton (*p* = 0.011) with a medium effect; poor sleepers (PSQI>5) had a significantly reduced WASO when sleeping in wool than in cotton (*p* = 0.047).

##### Wool vs polyester

In the same study (Chow et al., 2019) that showed a significant effect of sleepwear on SOL (*p* = 0.044), sleeping in wool sleepwear contributed to a lower SOL than sleeping in polyester sleepwear with no effect. Moreover, the sleep fragmentation index (SFI) was significantly lower when sleeping in wool (12.1 ± 4.2) than polyester (13.7 ± 4.4*) with a small effect (*p* = 0.005). When considering interaction effects with subgroups, for older adults (age > 65 years old), sleeping in wool significantly reduced SOL than sleeping in polyester (*p* = 0.011); for poor sleepers (PSQI > 5), sleeping in polyester significantly prolonged REM sleep latency compared with sleeping in wool (*p* = 0.036).

#### 3.5.2 Bedsheets

One study (Okamoto‐Mizuno et al., 2013) investigated the effect of bedsheets on sleep quality under warm conditions (29–30°C). The fibres included 100% linen and 100% cotton. The results showed that linen bedsheets promoted better sleep than cotton bedsheets (see below).

##### Linen bed sheet and pad vs cotton sheet + polyester bed pad

In the nap study (Okamoto‐Mizuno et al., 2013), conducted under mild humid heat conditions, compared 100% cotton sheet and pillowcases with 100% polyester bed pad and with 100% linen sheet, pillowcases, and bed pad on sleep quality. The condition with cotton sheets had a significantly increased number of awakenings and N1% compared with linen sheets and pillowcases with a large effect (*p* < 0.05). There were no significant differences for any other sleep variables.

#### 3.5.3 Duvets and quilts

Two studies investigated quilt materials and sleep quality (He et al., 2019; Shin et al., 2016). The materials as a filler included duck down, goose down, cotton, polyester, and wool. The studies were conducted under the ambient conditions of 11, 17, and 22°C. Goose down promoted longer SWS compared with cotton, and no significant differences were found between other materials.

##### Cotton vs duck down vs goose down

A study (He et al., 2019) showed quilt materials had a significant and large effect on sleep quality. Goose down promoted significantly longer SWS (N3%) compared with cotton with a large effect (*p* < 0.01), with no difference between duck down and cotton, or duck down and goose. No significant differences were shown for other PSG outcomes.

##### Wool vs polyester

From Shin's study (Shin et al., 2016), non‐significant differences were observed between wool and polyester quilts.

## 4 DISCUSSION

Six of the nine included studies reported that different fibre types that make up sleepwear or bedding significantly (*p* ≤ 0.05) affected sleep quality measured using various sleep outcomes with a medium to large effect (Chow et al., 2019; He et al., 2019; Lee et al., 2004; Okamoto‐Mizuno et al., 2013; Okamoto‐Mizuno et al., 2015; Shin et al., 2016). However, the relationship between sleep quality and the type of fibre used in sleepwear and bedding is intricate. Blended fibre sleepwear has shown potential advantages for certain groups. In cool environments, wool sleepwear has been found to aid sleep onset in adults, while in warm environments, it benefits older adults. Young adults experienced better sleep quality with linen sheets in hot conditions. Goose down duvets, under cool conditions, enhanced slow‐wave sleep in young adults. However, comparing different fibre types systematically is difficult due to the varied nature of the studies on sleep quality.

### 4.1 The performance of different sleepwear fibres on sleep quality

#### 4.1.1 Cotton vs blended materials

Despite five studies employing blended materials, comparisons between studies to derive a systematic sleep outcome is challenging for a diversity of methodological issues, namely (1) the blended fibre type differs, for example, Lee's study (Lee et al., 2004) used synthetic sources of polyester Healtha and polyolefin, while the other studies employed natural cellulose‐based fibres (Araujo et al., 2013; Nejedlá & Minařík, 2016; Utkun et al., 2015) or natural and synthetic materials (Okamoto‐Mizuno et al., 2015), (2) the target populations and study environment differed in that Lee et al. (Lee et al., 2004) studied healthy girls aged 12 ± 2 years in a sleep laboratory, while two studies conducted home studies in young children (7 years) with AD (Araujo et al., 2013), and or in infants (aged between 6 months to 12 months) (Utkun et al., 2015). A further two studies performed studies in young male adults (age < 30 years) in a sleep laboratory (Nejedlá & Minařík, 2016; Okamoto‐Mizuno et al., 2015). One study was conducted in warm conditions (Okamoto‐Mizuno et al., 2015), one in cool conditions (Nejedlá & Minařík, 2016), and three studies (Araujo et al., 2013; Lee et al., 2004; Utkun et al., 2015) did not report the ambient conditions.

It can only be concluded that for adolescent girls, sleepwear made of materials blended from synthetic sources (Lee et al., 2004) with the merits of super‐absorptive and fast‐drying capacities was effective in inducing more deep sleep and improving sleep quality compared with cotton sleepwear. This study (Lee et al., 2004) along with (Araujo et al., 2013; Utkun et al., 2015) were considered to have “good” methodological quality, although only study (Lee et al., 2004) yielded significant sleep changes with blended materials. The study suggests that blended fibres, with superior absorption, quick‐drying properties, and lower thermal conductivity than cotton, enhance sleep quality, particularly SWS when the body temperature was usually lower than other sleep stages (Szymusiak, 2018), in girls aged 12 ± 2 years, possibly by better maintaining body temperature. However, the ambient temperature during the study was not reported. No differences in sleep outcomes were observed between cotton and other blended sleepwear in infants, children, and young men.

In sum, these diverse studies underscore the intricate interplay between fibre composition, weave structure, and their combined influence on sleep outcomes. The findings highlight both similarities and disparities in sleep quality between cotton and blended materials, contextualising the multifaceted factors influencing sleep experiences within varying populations.

#### 4.1.2 Pure materials

Two studies (Chow et al., 2019; Shin et al., 2016), both considered to have “good” methodological quality, investigated the sleep effect among sleepwear made of cotton, polyester, and Merino wool, with significant differences reported. Shin's study (Shin et al., 2016) was conducted among young (25 ± 7 years) healthy adults at 17 and 22°C, while Chow's study (Chow et al., 2019) was conducted among relatively older adults (60 ± 6 years) at 30°C. The comparisons between materials are discussed below.

##### Cotton vs wool

Significant sleep benefits were observed with wool compared with cotton, including a shortened sleep onset in young adults in cooler conditions (17°C) (Shin et al., 2016) and in older adults in warmer conditions (30°C) (Chow et al., 2019). Wool also led to decreased N2% and increased N3% sleep stages in young adults at 17°C but not at 22°C (Shin et al., 2016). Interestingly, cotton sleepwear showed a greater N3% at 22°C (Shin et al., 2016). These findings indicated that wool sleepwear performed better under cooler conditions, while cotton sleepwear would be more suitable for warmer thermal conditions in young adults. In Chow's study (Chow et al., 2019) that compared cotton sleepwear with wool sleepwear, older adults and poor sleepers (with a PSQI >5) benefitted more from wool, showing a shorter SOL, lower WASO, and shorter REM latency. This suggests that these individuals may prefer sleeping at a higher ambient temperature for more favourable thermal comfort (Giamalaki & Kolokotsa, 2019; Wong et al., 2009) which wool sleepwear can provide due to its superior insulation and moisture transport properties (Ukponmwan, 1993). Additionally, wool sleepwear can help to regulate the body temperature and prevent overheating or getting too cold by trapping air and moisture, creating a favourable microclimate between the skin and garment (Iqbal, 2021).

In summary, wool sleepwear is favourable for cooler conditions, older populations, and poorer sleepers for a faster sleep onset and more consolidated sleep. For healthy young adults under normal ambient temperatures, cotton sleepwear would be better for a deeper sleep.

##### Cotton vs polyester

Sleepwear made of cotton and polyester was compared in one study (Chow et al., 2019) in older adults under warm conditions but no significant differences in sleep outcomes were reported.

##### Wool vs polyester

In this comparison, one study (Chow et al., 2019) found that older adults who slept in wool under warmer conditions experienced significant improvements in their sleep. Specifically, they had a shortened SOL and a decrease in SFI, indicating fewer disruptions during sleep compared with polyester. Furthermore, among older adults with poor sleep quality, those who slept in wool had decreased REM latency, meaning they entered the REM sleep stage quicker compared with those who slept in polyester. However, in young adults, non‐significant differences were observed between wool and polyester quilts under cool and comfortable conditions (Utkun et al., 2015). These findings suggested that wool sleepwear performed better than polyester sleepwear by contributing to a shorter sleep onset and maintaining a less fragmented sleep, especially for older adults in warmer conditions.

Taken together, material properties such as weight and thermal resistance can determine the thermo‐physiological wear comfort and skin sensation wear comfort (Rechtschaffen & Kales, 1968; Zaki et al., 2021) and can in turn affect sleep quality. In Chow's study (Nejedlá & Minařík, 2016), the weight and thickness of the wool sleepwear lies between cotton and polyester sleepwear, while the thermal resistance values were similar. The higher moisture buffering of wool sleepwear (9.9 KJ·m^−2^) compared with polyester (0.6 KJ·m^−2^) or cotton (6.9 KJ·m^−2^) (Pan et al., 2012) potentially contributed to a faster sleep onset, which was associated with a fall in core body temperature and a rise in distal skin temperature (Fanger, 1970; Gagge et al., 1967; Lan et al., 2017). Sleeping in wool also showed the lowest SFI compared with cotton and polyester sleepwear. A lower SFI reflected fewer stage shifts and less thermal stress under hot humid conditions (Hosseini Ravandi & Valizadeh, 2011), which may be linked to the beneficial moisture transfer and the wicking properties of wool. While in Shin's study (Utkun et al., 2015), the wool sleepwear was a little heavier and thinner than the cotton sleepwear, with a higher air permeability, lower vapour resistance, and similar thermal resistance value compared with cotton sleepwear. A previous study (Lan et al., 2017) showed that slight warming of proximal skin in the comfortable range would decrease SOL and enhance SWS. The wool sleepwear might perform better in keeping the proximal skin warm. [Correction added on 20 May 2024, after first online publication: The numbering of subsections 4.1.3, 4.1.4 and 4.1.5 have been removed. All 3 subsections are now included under 4.1.2.].

### 4.2 The performance of different bedsheet materials on sleep quality

Only one study (Okamoto‐Mizuno et al., 2013) investigated the effect of bedsheets on sleep quality under a warm/hot condition, which compared a composite of linen (hemp) bedsheets, bed pads, and pillowcases with a composite of cotton sheets, polyester bed pads, and cotton pillowcases. The results indicated that the linen composite promoted a better sleep through a significantly shorter W%, N1%, and fewer awakenings compared with cotton composite. Given the bed pads were different between conditions, it is difficult to establish whether the bedsheet fibre type or bed pad played a more important role in this situation.

### 4.3 The performance of different duvets and quilts materials on sleep quality

Based on the studies reviewed, only He's study (He et al., 2019) reported a significant impact of duvet material on sleep quality. Duvets filled with goose down promoted the longest N3% compared with duvets filled with cotton when sleeping under a cool condition (11°C). Duvets filled with duck down also showed a longer N3% compared with that with cotton but non‐significant differences reported. This difference may be explained by the higher insulation value and lower thermal conductivity of feather down compared with cotton, which would have created a thermal comfort bed micro‐climate for people under a cool condition. Conversely, an uncomfortable cool condition would increase muscle activity, stimulate wakefulness, and improve the frequency of the arousals or stage transition from SWS to shallow sleep (Pan et al., 2012). Meanwhile, there were no significant differences found between wool and polyester quilts under normal ambient condition (17 and 22°C) (Shin et al., 2016).

### 4.4 Limitations of the included studies

Although the studies included in this review shed light on the potential impact of sleepwear and bedding materials on sleep quality, there are some limitations that should be noted. Firstly, the sample sizes in some studies were relatively small, like Nejedlá's study (Nejedlá & Minařík, 2016) that included one participant. Four studies (He et al., 2019; Lee et al., 2004; Okamoto‐Mizuno et al., 2013; Utkun et al., 2015) had sample sizes of <10 participants, which may limit the generalisability of the findings. However, by comparison, the studies by Shin et al. (Shin et al., 2016) and Chow et al. (Chow et al., 2019) reported a sample size of *N* = 17 and *N* = 36, respectively. Secondly, the studies employed different materials under different ambient conditions. Indeed, Xu and Lian highlighted the importance of the relationship between thermal environment, body temperature, human's thermal comfort and sleep quality (Xu & Lian, 2023). Since humans are sensitive to temperature differences (Fanger, 1970) which influence thermal comfort and sleep propensity and quality (Gagge et al., 1967; Lan et al., 2017), sleep outcomes from different studies are not directly comparable. Some studies did not report the temperature conditions (Araujo et al., 2013; Lee et al., 2004; Nejedlá & Minařík, 2016) or fibre material properties (Araujo et al., 2013; Nejedlá & Minařík, 2016; Zaki et al., 2021), making it difficult to draw definitive conclusions about the effects of specific materials or conditions on sleep. However, other studies (Chow et al., 2019; He et al., 2019; Lee et al., 2004; Okamoto‐Mizuno et al., 2013; Okamoto‐Mizuno et al., 2015; Shin et al., 2016; Utkun et al., 2015) clearly presented the properties of the fibres. Finally, some studies had limitations in their methodology.

Specifically, Utkun's study (Utkun et al., 2015) used fibres with different structures and yarns, thus making it difficult to determine the specific characteristics of the fibres and to compare the fibre properties. Sleep duration was reported by the mother, which may introduce biased reporting and errors.

Araujo's study (Araujo et al., 2013) did not report the material properties of the sleepwear and did not conduct objective sleep measurement in the experiment. Furthermore, it is worth noting that there was a difference in sleep disturbances between the blended and cotton sleepwear groups at baseline (day 0), with values of 5.2 ± 2.3 and 4.7 ± 2.4, respectively. This finding raises the possibility of introducing bias and reducing the accuracy and reliability of the study's results.

Okamoto‐Mizuno's studies (Okamoto‐Mizuno et al., 2013; Okamoto‐Mizuno et al., 2015) investigated the effects of different sleepwear and beddings under hot and humid conditions during daytime naps, which may not be representative of night‐time sleep, as a human's body temperature would change according to the thermoregulatory system.

Nejedlá's study (Nejedlá & Minařík, 2016), as mentioned earlier, is considered to have a high risk of bias due to the small sample size and lack of proper statistical analysis. This paper did not report any material properties or the bedroom environment conditions.

### 4.5 Limitation of this review and implications for future research

This review has several limitations that should be considered when interpreting the findings. Firstly, the inclusion criteria only considered papers with English abstracts, which may have resulted in the exclusion of relevant studies published in other languages. Additionally, two of the included papers (Okamoto‐Mizuno et al., 2013; Okamoto‐Mizuno et al., 2015) were translated using Google Translate, which may have led to some misinterpretation or omission of key information. Furthermore, publication bias may have influenced the results, as studies with statistically significant results are more likely to be published.

Moreover, due to the heterogeneity of the studies included in this review, such as varying designs, materials, and outcome measures, it was not possible to conduct a meta‐analysis, thus limiting the generalisability of the findings. Most studies were conducted in laboratory settings, which may not reflect real‐world sleeping conditions.

Sleep quality can be affected by the performance of different fibre types (along with their material properties, fibre weave types, and blending) in different ambient conditions. Material properties such as weight and thermal resistance can determine the thermo‐physiological wear comfort and skin sensation wear comfort (Hosseini Ravandi & Valizadeh, 2011; Saville, 1999) and can in turn affect sleep quality. Other physiological factors such as sex, age, and metabolic rates can also impact the interaction with microclimate and sleep quality (Kayabekir, 2019; Okamoto‐Mizuno & Tsuzuki, 2010). The human metabolic processes generate heat and moisture, which interact with the clothing with respect to its dissipation and affect comfort (Bhatia & Malhotra, 2016). These factors were not considered in this review.

Future research should include larger and more diverse samples, standardised study designs with specific types of fibres for sleepwear/bedding, and objective outcome measures of sleep (using polysomnography and actigraphy) to provide a more comprehensive understanding of the effects of sleepwear and bedding materials on sleep quality. The authors recommend testing the difference between two ensembles (for example): one with cotton sleepwear, bedding, and pillowcases, and another with wool sleepwear, bedding, and pillowcases.

## 5 CONCLUSION

Overall, the reviewed studies suggest that different types of sleepwear, bedsheets, and duvet materials can affect sleep outcomes, and selecting appropriate materials for sleepwear, bedsheets, and duvets can have a positive impact on sleep quality. However, based on the limited evidence from this review, it is hard to draw an overall conclusion. Some points can be drawn from the comparison of subgroups. For sleepwear, wool sleepwear appears to be the most beneficial for promoting sleep quality compared with cotton or polyester sleepwear, while sleepwear made of materials blended from synthetic sources was effective in inducing more deep sleep and improving sleep quality compared with cotton sleepwear for adolescent girls. There were no significant differences reported or no evidence for other fibre or condition. For bedding, as only one study was included in this review, which showed under hot conditions, linen promoted less W%, N1%, and awakening in healthy young men compared with a combination of cotton and polyester bedding, no conclusion can be drawn for this section for the lack of evidence. For the duvets, under cool conditions, duvets filled with goose down were preferable to cotton‐filled duvets, while there was no significant difference between cotton and duck down. Meanwhile, under normal temperatures, there was no significant difference found between wool and polyester quilts either. However, the heterogeneity of the studies included, and the limitations of this review indicate a need for more standardised research with larger and more diverse samples to fully understand the effects of sleepwear and bedding fibre materials on sleep quality. Nonetheless, the findings of this review provide valuable insights for individuals seeking to improve their sleep quality and for companies designing sleep products.

## AUTHOR CONTRIBUTIONS

**Xinzhu Li:** Writing – original draft. **Mark Halaki:** Writing – review and editing. **Chin Moi Chow:** Writing – review and editing.

## CONFLICT OF INTEREST STATEMENT

CMC received research funding from Australian Wool Innovation Ltd (AWI). XL received a research scholarship from Australian Wool Innovation Ltd (AWI). MH reports no conflicts of interest in this work.

## Data Availability

Data sharing is not applicable to this article as no new data were created or analyzed in this study.
